# Haemodynamic effects of clonidine in an ovine model of severe sepsis with septic acute kidney injury

**DOI:** 10.1186/cc13604

**Published:** 2014-03-17

**Authors:** P Calzavacca, Y Lankadeva, L Booth, S Bailey, M Bailey, R Bellomo, CN May

**Affiliations:** 1 AO Melegnano, PO Uboldo, Cernusco sul Naviglio, Italy; 2 Melbourne University, Parkville, Australia; 3 Monash University, Clayton, Australia; 4 Austin Health, Heidelberg, Australia

## Introduction

In sepsis, sympathetic nerve activity is increased, which helps maintain arterial pressure in the face of nitric oxide-induced vasodilatation. Accordingly, we investigated the haemodynamic effects of the centrally acting α-adrenoceptor agonist clonidine in an ovine model of severe sepsis.

## Methods

A prospective interventional blinded crossover study in 12 Merino ewes with cardiac and renal flow probes implanted to continuously measure cardiac output and renal blood flow. Arterial pressure was continuously monitored and blood and urine samples were taken. After 24 hours of control, sepsis was induced by an intravenous bolus and continuous infusion of live *Escherichia coli *for 32 hours. After 24 hours of sepsis, animals were randomly and blindly allocated to vehicle infusion or clonidine (1 μg/ml/kg/minute) for 8 hours. The *E. coli *infusion was then discontinued, gentamycin 150 mg given i.m. and the animals were followed for 16 hours during recovery. The animals that survived were crossed over to the alternative treatment 2 weeks later.

## Results

**Figure 1:**
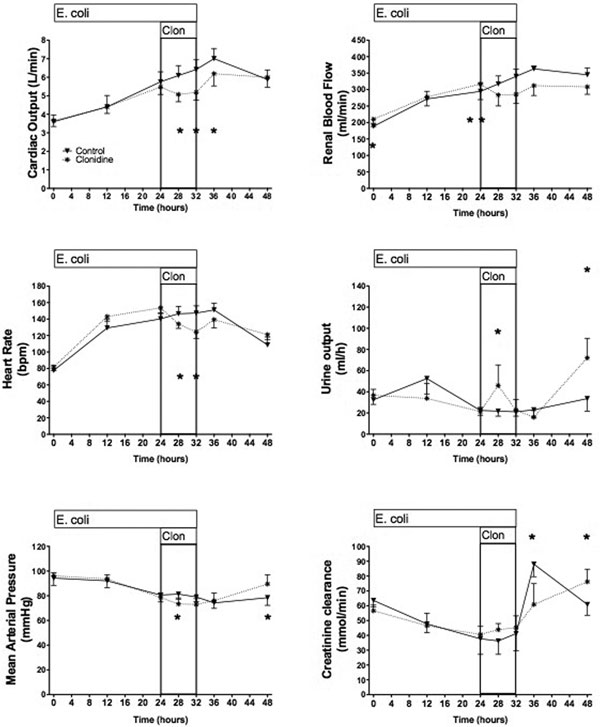
Haemodynamic and renal effects of clonidine in established sepsis.

Complete data were collected on eight animals/group, three animals died/group. Hyperdynamic sepsis with hypotension and acute kidney injury of similar degree developed in the two groups. Haemodynamic and renal effects of clonidine are shown in Figure 1.

## Conclusion

In ovine hyperdynamic sepsis, clonidine transiently increased urine output without affecting creatinine clearance.

